# Correlation analysis of coronary artery tortuosity and calcification score

**DOI:** 10.1186/s12893-022-01470-w

**Published:** 2022-02-23

**Authors:** Min Li, Zhen-Wei Wang, Li-Juan Fang, Shou-Quan Cheng, Xin Wang, Nai-Feng Liu

**Affiliations:** Department of Cardiology, Zhongda Hospital, School of Medicine, Southeast University, Nanjing, 210009 People’s Republic of China

**Keywords:** Coronary artery tortuosity, Coronary artery calcification, Atherosclerosis, Calcium score

## Abstract

**Background:**

Coronary artery tortuosity (CAT) is regarded as a variation of vascular anatomy, and its relationship with coronary artery calcification (CAC) score is still not well clarified. Studying the correlation between coronary artery calcification scores and CAT to determine specific prevention and intervention populations seems to have more meaningful.

**Methods:**

The study is a cross-sectional retrospective study, including 1280 patients. CAT is defined as the presence of at least three consecutive curvatures of more than 45°measured during systole or diastole of a major epicardial coronary artery. Multivariable regression analysis was used to adjust the clinical parameters directly affecting CAT.

**Results:**

Of these individuals, 445 (35%) were evaluated having CAT, of which females are higher than males (59.1% vs. 40.9%). Moderate CAC score (101–400) (odds ratio (OR) 1.49, 95% confidence interval [95%CI] 1.05–2.10, *P* = 0.025) revealed significantly associated with CAT on univariable analysis. However, multivariable analysis after adjusting for confounding factors only indicated that CAT was positively correlated with female (OR 1.68, 95%CI 1.30–2.17, *P* < 0.001), hypertension (OR 1.35, 95% CI 1.04–1.75, *P* = 0.024), and age (OR 1.02, 95% CI 1.01–1.03, *P* = 0.001), while was negatively associated with body mass index (BMI) 24–27.9(OR 0.76, 95% CI 0.58–1.00, *P* = 0.044), and BMI > 28 (OR 0.46, 95% CI 0.31–0.68, *P* < 0.001). Further analysis stratified by gender showed that compared with non-CAT, CAT was significantly linked with moderate CAC score (OR 1.79, 95% CI 1.00–3.20, *P* = 0.048), hypertension (OR 1.54, 95% CI 1.07–2.22, *P* = 0.021), and high-density lipoprotein (HDL) (OR 1.86, 95% CI 1.07–3.24, *P* = 0.028), while was negatively related to BMI > 28 (OR 0.51, 95% CI 0.31–0.84, *P* = 0.008) in female patients.

**Conclusions:**

CAT is more likely to be found in females, connected with hypertension, age, and BMI. No significant correlation is found between the presence of tortuosity and calcium score or diameter stenosis on multivariable analysis. Whereas the CAT is associated with moderate CAC score in correlation analysis when women are selected as the main group.

**Supplementary Information:**

The online version contains supplementary material available at 10.1186/s12893-022-01470-w.

## Introduction

Vascular calcification is the active deposition process of bone-specific hydroxyapatite crystallization in the blood vessel wall caused by multiple pathogenic factors [1]. Vascular calcification is prevalent and is related to many diseases such as aging, diabetes, atherosclerosis and chronic kidney disease. Long-term vascular calcification can cause vessel stiffness and reduced compliance, leading to adverse cardiovascular events [2]. Raggi et al. documented that the progression of coronary calcification was more common in patients who had clinical coronary events compared with event-free subjects [3]. This view has also been confirmed by a multi-ethnic study of atherosclerosis conducted by Budoff et al., suggesting that vascular calcification is significantly associated with all-cause mortality and atherosclerotic plaque rupture [4]. The assessment of CAC progression makes it possible to monitor the progression of coronary plaque burden and evaluate the modification of risk factors and the success of medical treatments [5, 6]. Computed tomography angiography (CTA) provides a unique diagnostic value in the estimation of CAC progression, and it can be detected, located and quantified, which is of great significance to the risk assessment of coronary artery disease [7].

Furthermore, CAT is a common phenomenon found in coronary angiography [8]. The etiology and clinical significance of CAT remain unclear [9]. Preliminary evidence suggests that it is associated with various diseases, including aging, hypertension, reversible myocardial reperfusion injury, spontaneous coronary artery dissection, fibromuscular dysplasia and connective tissue diseases [10]. Nevertheless, the correlation between CAT and atherosclerosis has been controversial, and few types of research have revealed its correlation with calcification score. It seems to be more beneficial to study the correlation between CAC score and vascular tortuosity in order to identify specific prevention and intervention populations.

## Patients and methods

### Clinical study design

This is a cross-sectional retrospective study registered in the China Clinical Trial Registration Center (ChiCTR1800020259). The study included patients with suspected coronary artery disease and stable hemodynamics who underwent coronary CTA examinations using 320-row-detector dynamic volume CT from January 1, 2020 to March 9, 2021.The patients with a past history of coronary stent implantation, coronary artery bypass graft, prosthetic valve or pacemaker implantation, pregnancy, chronic renal insufficiency, and those presenting any contraindication for iodinated compounds were excluded.

### CT angiography

Coronary CTA images were reviewed by two experienced radiologists who were blind to all clinical profiles of the participants. CAC score was calculated by dedicated software and quantified as the Agatston score [11]. According to the ACCF/AHA 2007 clinical expert consensus, the Agatston score was divided into 4 levels (0, 1–100, 101–400, > 400) [12]. In addition, the severity of coronary stenosis was classified as up to 0, 1–24%, 25–49%, 50–69%, 70–100%.

### Biochemical indexes

Venous blood samples were collected from all participants after a 12-h overnight fast. Biochemical parameters were determined in the Department of Clinical Laboratory, Zhong Da hospital affiliated Southeast University.

### Risk factors

Hypertension was defined as systolic blood pressure ≥ 140 mmHg or/and diastolic blood pressure ≥ 90 mmHg, or taking antihypertensive drugs. Diabetes was diagnosed according to the WHO criteria. Smokers were defined as those smoking in the past 1 month.

### Statistical analysis

IBM SPSS software (version 24.0, SPSS, Inc.) was used for statistical analysis. All data were accomplished a normal distribution test before analysis. Continuous variables were expressed as the mean ± standard deviation (SD) if normally distributed, otherwise as median (interquartile range); in addition, comparisons between groups performed by the *t* test if normally distributed or the Mann–Whitney test if not normally distributed. Categorical variables were described as percentages and compared by χ^2^ testing. Multivariable logistic regression was used to assess the association of CAT with calcification score. The final model was adjusted for sex, age, BMI, hypertension, diameter stenosis, uric acid, TG, and HDL. Univariable and multivariable analysis were performed to identify clinical parameters directly affecting coronary tortuosity. All tests were 2-sided and *P* values < 0.05 were considered statistically significant.

## Results

### Baseline characteristics

**Fig. 1:**
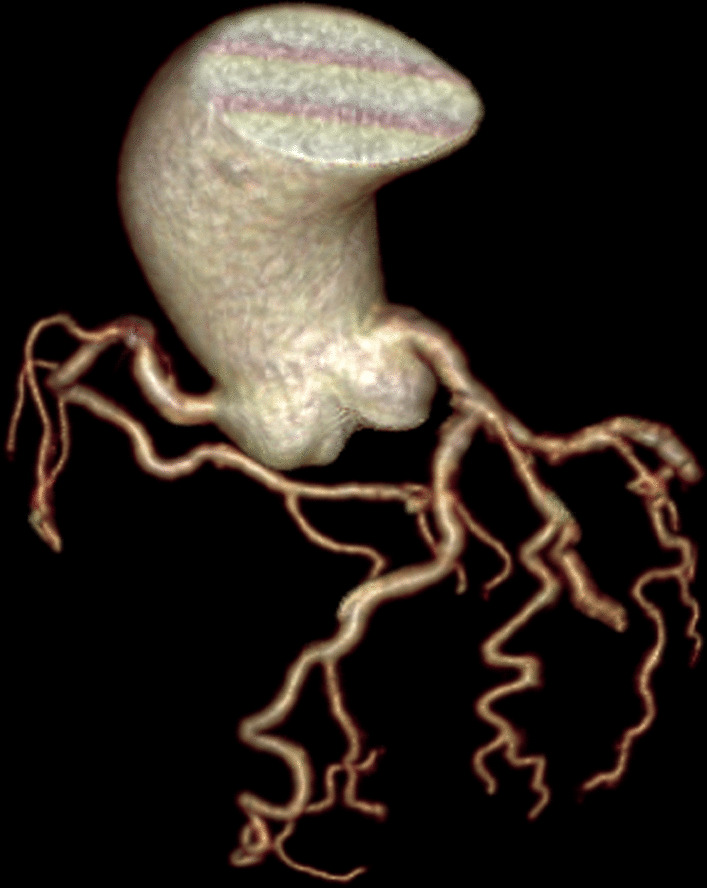
CTA shows tortuous coronary

Baseline characteristics of participants in the study were presented (Table 1). A total of 1280 subjects were included in the analysis, of whom 445 (35%) were CAT group (Fig. 1), and 835 (65%) were N-CAT group. The mean ± SD age of the participants was 62.3 ± 12.7 years. All variables, except gender (*P* < 0.001), age (*P* < 0.001), BMI (*P* < 0.001), hypertension (*P* = 0.014), uric acid (*P* = 0.018), triglycerides (TG) (*P* = 0.047), and high-density lipoprotein (HDL) (*P* < 0.001) were similar in both groups.

**Table 1:** General characteristics of patients included in the study

Characteristic	Total	N-CAT group	CAT group	*p*-value
Participants (n)	1280	835 (65%)	445 (35%)	
Gender				
Male (n)	658 (51.4%)	476 (57%)	182 (40.9%)	< 0.001
Female (n)	622 (48.6%)	359 (43%)	263 (59.1%)	
Age (years)	62.3 ± 12.7	61.1 ± 12.9	65.2 ± 11.8	< 0.001
BMI (kg/m^2^)				
18.5–23.9	412 (31.2%)	238 (28.5%)	174 (39.1%)	< 0.001
24–27.9	635 (49.6%)	418 (50.1%)	217 (48.8%)	
> 28	233 (18.2%)	179 (21.4%)	54 (12.1%)	
Myocardial bridge	555 (43.4%)	349 (41.8%)	206 (46.3%)	0.122
Diameter stenosis				
No stenosis	518 (40.5%)	351 (42%)	167 (37.5%)	0.204
Slight stenosis	207 (16.2%)	134 (16.1%)	73 (16.4%)	
Mild stenosis	242 (18.9%)	153 (18.3%)	89 (20%)	
Moderate stenosis	182 (14.2%)	107 (12.8%)	75 (16.9%)	
Severe stenosis	131 (10.2%)	90 (10.8%)	41 (9.2%)	
Agatston score				
0	608 (47.5%)	411 (49.2%)	197 (44.2%)	0.111
1–100	342 (26.7%)	226 (27.1%)	116 (26.1%)	
101–400	173 (13.5%)	101 (12.1%)	72 (16.2%)	
> 400	157 (12.3%)	97 (11.6%)	60 (13.5%)	
Smoking	189 (14.8%)	131 (15.7%)	58 (13%)	0.202
Hypertension	758 (59.2%)	474 (56.8%)	284 (63.8%)	0.014
Diabetes	266 (20.8%)	178 (21.3%)	88 (19.8%)	0.517
Biochemical indexes				
Uric Acid (μmol/l)	341 ± 103.2	346.1 ± 104.1	331.6 ± 101	0.018
TG (mmol/l)	1.6 ± 1.2	1.7 ± 1.2	1.5 ± 1.2	0.047
CHOL (mmol/l)	4.5 ± 1.0	4.5 ± 1.0	4.6 ± 1.1	0.475
HDL (mmol/l)	1.3 ± 0.3	1.3 ± 0.3	1.4 ± 0.3	< 0.001
LDL (mmol/l)	2.5 ± 0.8	2.6 ± 0.8	2.5 ± 0.7	0.496
ApoA1(g/l)	1.2 ± 0.3	1.2 ± 0.3	1.2 ± 0.3	0.582
ApoB (g/l)	0.9 ± 0.2	0.9 ± 0.2	0.9 ± 0.2	0.633
LPa (mg/l)	124 (59–296)	118 (58–274)	138 (61–328)	0.117

### No correlation between CAT and CAC score

The correlation between CAT and CAC score was analyzed (Table 2). Individuals with an Agatston score from 101 to 400 were more likely to coexist with CAT than those with an Agatston score 0 (OR 1.49, 95% CI 1.05–2.10, *P* = 0.025) in unadjusted analysis (Table 3 and Additional file 1: Table S1). However, on multivariable analysis, CAT was not significantly associated with Agatston score after adjusting for covariates (sex, age, BMI, hypertension, diameter stenosis, uric acid, TG, and HDL) (Table 2).

**Table 2:** Associations between CAT and CAC score

	Model 1		Model 2		Model 3	
	Odds ratio (95% CI)	*p*-value	Odds ratio (95% CI)	*p*-value	Odds ratio (95% CI)	*p*-value
Agatston score						
0	Ref		Ref		Ref	
1–100	0.97 (0.72–1.31)	0.831	0.91 (0.61–1.35)	0.635	0.93 (0.63–1.38)	0.712
101–400	1.35 (0.92–1.97)	0.125	1.37 (0.83–2.28)	0.224	1.40 (0.84–2.32)	0.200
> 400	1.01 (0.67–1.52)	0.957	1.16 (0.66–2.06)	0.605	1.22 (0.69–2.17)	0.500
*p* for trend	0.375		0.312		0.314	

Model 1 was adjusted for sex, age and BMI

Model 2 was adjusted for sex, age, BMI, hypertension and diameter stenosis

Model 3 was adjusted for sex, age, BMI, hypertension, diameter stenosis, uric acid, TG, and HDL

**Table 3:** Univariable and multivariable analysis of which factors are related to CAT

Variable	Univariable analysis		Multivariable analysis	
	Odds ratio (95% CI)	*p*-value	Odds ratio (95% CI)	*p*-value
Gender				
Male	Ref		Ref	
Female	1.92 (1.52–2.42)	< 0.001	1.68 (1.30–2.17)	< 0.001
Age (years)	1.03 (1.02–1.04)	< 0.001	1.02 (1.01–1.03)	0.001
BMI (kg/m^2^)				
18.5–23.9	Ref		Ref	
24–27.9	0.71 (0.55–0.92)	0.009	0.76(0.58–1.00)	0.044
> 28	0.41 (0.29–0.59)	< 0.001	0.46(0.31–0.68)	< 0.001
Diameter stenosis				
No stenosis	Ref		N/A	
Slight stenosis	1.15 (0.82–1.61)	0.434		
Mild stenosis	1.22 (0.89–1.68)	0.218		
Moderate stenosis	1.47 (1.04–2.09)	0.029		
Severe stenosis	0.96 (0.63–1.45)	0.837		
Agatston score				
0	Ref		N/A	
1–100	1.07 (0.81–1.42)	0.633		
101–400	1.49 (1.05–2.10)	0.025		
> 400	1.29 (0.89–1.86)	0.170		
Hypertension	1.34 (1.06–1.70)	0.015	1.35(1.04–1.75)	0.024
Uric Acid (μmol/l)	0.99 (0.97–1.00)	0.019	1.00 (0.99–1.00)	0.922
TG (mmol/l)	0.90 (0.81–1.00)	0.049	0.98 (0.88–1.10)	0.696
HDL (mmol/l)	2.02 (1.38–2.96)	< 0.001	1.47 (0.97–2.23)	0.068

### Factors affecting the incidence of CAT

On univariable analysis, female was more likely to have CAT than male (OR 1.92, 95% CI 1.52–2.42, *P* < 0.001) (Table 3 and Additional file 1: Table S1). The increase of age was also significantly related to CAT (OR 1.03, 95% CI 1.02–1.04, *P* < 0.001). Participants with hypertension were more possible than those without to be CAT (OR 1.34, 95% CI 1.06–1.70, *P* = 0.015). Compared with patients without lumen stenosis, patients with moderate lumen stenosis were associated with CAT (OR 1.47, 95% CI 1.04–2.09, *P* = 0.029). In addition, overweight (BMI 24–27.9) (OR 0.71, 95% CI 0.55–0.92, *P* = 0.009) and obese patients (BMI > 28) (OR 0.41, 95% CI 0.29–0.59, *P* < 0.001) were less likely to coexist with CAT than normal weight patients (BMI 18.5–23.9) on univariable analysis.

On multivariable analysis, after adjusting for the parameters that affect CAT, female (OR 1.68, 95% CI 1.30–2.17, *P* < 0.001), age (OR 1.02, 95% CI 1.01–1.03, *P* = 0.001), and hypertension (OR 1.35, 95% CI 1.04–1.75, *P* = 0.024) remained significantly associated with CAT (Table 3). Furthermore, those who were overweight (OR 0.76, 95% CI 0.58–1.00, *P* = 0.044) and obese (OR 0.46, 95% CI 0.31–0.68, *P* < 0.001) were less likely to have CAT than those with normal weight.

### Moderate calcification score is related to CAT in female patients

In order to explore the correlation between coronary tortuosity and CAC score in special populations, gender was stratified and further analyzed. Univariate analysis revealed that in female patients, compared with patients with an Agatston score of 0, patients with a high Agatston score were significantly associated with CAT (1–100, OR 1.49, 95% CI 1.01–2.18, *P* = 0.042; 101–400, OR 2.36, 95% CI 1.36–4.07, *P* = 0.002; > 400, OR 1.82, 95% CI 1.07–3.11, *P* = 0.028), while this correlation was not found in male patients (Table 4 and Additional file 1: Table S2). However, on multivariate analysis, CAT in female patients was only significantly associated with moderate Agatston score after adjusting for age and BMI (OR 1.94, 95% CI 1.09–3.45, *P* = 0.024), and after full adjustment (age, BMI, hypertension, and HDL) (OR 1.80, 95% CI 1.00–3.22, P = 0.048). Moreover, CAT in female patients was positively correlated with hypertension (OR 1.54, 95% CI 1.07–2.22, *P* = 0.021) and HDL (OR 1.86, 95% CI 1.07–3.24, *P* = 0.028), and negatively correlated with obesity (OR 0.51, 95% CI 0.31–0.84, *P* = 0.008), while it in male patients was positively associated with age (OR 1.03, 95% CI 1.02–1.04, *P* < 0.0001) and negatively associated with overweight (OR 0.72, 95% CI 0.56–0.94, *P* = 0.014) and obesity (OR 0.46, 95% CI 0.32–0.66, *P* < 0.0001) (Tables 5 and 6).

**Table 4:** Associations between CAT and CAC score in female

	Model 1		Model 2		Model 3	
	Odds ratio (95% CI)	*p*-value	Odds ratio (95% CI)	*p*-value	Odds ratio (95% CI)	*p*-value
Agatston score						
0	Ref		Ref		Ref	
1–100	1.49 (1.01–2.18)	0.042	1.29 (0.89–1.93)	0.226	1.29 (0.86–1.95)	0.224
101–400	2.36 (1.36–4.07)	0.002	1.94 (1.09–3.45)	0.024	1.80 (1.00–3.22)	0.048
> 400	1.82 (1.07–3.11)	0.028	1.34 (0.74–2.43)	0.332	1.33 (0.72–2.44)	0.358
*p* for trend	0.004		0.140		0.226	

Model 1 was unadjusted

Model 2 was adjusted for age and, BMI

Model 3 was adjusted for age, BMI, hypertension, and HDL

**Table 5:** Univariable and multivariable analysis of parameters directly affecting for CAT in female

Variable	Univariable analysis		Multivariable analysis	
	Odds ratio (95% CI)	*p*-value	Odds ratio (95% CI)	*p*-value
Age (years)	1.03 (1.01–1.04)	< 0.001	1.01 (0.99–1.03)	0.126
BMI (kg/m^2^)				
18.5–23.9	Ref		Ref	
24–27.9	0.75 (0.53–1.07)	0.111	0.73 (0.51–1.06)	0.094
> 28	0.49 (0.31–0.81)	0.005	0.51 (0.31–0.84)	0.008
Agatston score				
0	Ref		Ref	
1–100	1.49 (1.01–2.18)	0.042	1.29 (0.86–1.95)	0.224
101–400	2.36 (1.36–4.07)	0.002	1.79 (1.00–3.20)	0.048
> 400	1.82 (1.07–3.11)	0.028	1.33 (0.72–2.44)	0.358
Hypertension	1.68 (1.21–2.33)	0.002	1.54 (1.07–2.22)	0.021
HDL (mmol/l)	1.88 (1.12–3.18)	0.018	1.86 (1.07–3.24)	0.028

**Table 6:** Univariable and multivariable analysis of factors directly affecting for CAT in male

Variable	Univariable analysis		Multivariable analysis	
	Odds ratio (95% CI)	*p*-value	Odds ratio (95% CI)	*p*-value
Age (years)	1.02 (1.00–1.03)	0.002	1.03 (1.02–1.04)	< 0.0001
BMI				
18.5–23.9	Ref		Ref	
24–27.9	0.77 (0.52–1.13)	0.175	0.72 (0.56–0.94)	0.014
> 28	3.62 (0.21–0.64)	< 0.0001	0.46 (0.32–0.66)	< 0.0001
Diameter stenosis				
No stenosis	Ref		Ref	
Slight stenosis	1.61 (0.98–2.64)	0.06	1.05 (0.74–1.50)	0.769
Mild stenosis	1.67 (1.02–2.74)	0.041	0.95 (0.68–1.34)	0.782
Moderate stenosis	1.51 (0.90–2.52)	0.116	1.05 (0.72–1.52)	0.819
Severe stenosis	1.01 (0.56–1.82)	0.937	0.67 (0.43–1.04)	0.071

## Discussion

In this study of 1280 participants, it is documented that age and hypertension may be the main factors for the occurrence of CAT, which is consistent with the results of other researchers [8, 13]. It should be understood that arteries are usually straight tubes that can effectively transport blood to remote organs. However, due to developmental abnormalities or vascular disease, arteries may be tortuous. The maintenance of arterial stability in vivo mainly depends on a certain degree of axial tension, pressure and traction, and the retraction force produced by elastin which can resist pressure and traction [14–17]. These forces are essential in preventing tortuosity. The age-related coronary tortuosity is related to the degradation and decrease of elastin [18–20]. In addition, the axial tension may decrease with aging [17, 21]. Hypertension or increased blood flow also contributes to artery tortuosity associated with elastin degradation and fragmentation, which has been considered the cause of vessel lengthening [22, 23].

The results of the study on the correlation between gender differences and CAT are also consistent with the findings of other current works [13, 24]. It also explains why women often show symptoms of chest pain, but have better angiographic results being normal coronary arteries or less severe disease than men [24]. In addition, the uniqueness of this work is that it shows the correlation between BMI and CAT. Compared with people with normal weight, overweight or obesity is negatively correlated with CAT. Previous studies have reported that the incidence of tortuosity increased as the heart size and mass decreased [25]. Based on this theory, we speculate that long-term overweight and obesity are accompanied by an increase in the thickness of epicardial adipose tissue, which may have a certain impact on the shape and length of blood vessels.

We did not find a negative correlation between CAT and atherosclerosis described by Li et al. [8], which may be due to different methods used to calculate the coronary tortuosity. Beyond that, our study displays no correlation between Agatston score and CAT except in the female population. Result contrasts with research by Tahlawi et al., who revealed that CAT is associated with Agatston score even in the absence of significant obstructive lesion [9]. This may be due to the large population cohort and detailed stratification of calcification score included in our study. Even so, the CAC score is highly specific in atherosclerosis [26]. However, the result that compared with patients without vascular calcification, those with moderate calcification score are associated with CAT in females is discovered. It is known that severe intravascular calcification can lead to the stiffness and rigidity of the vascular wall, which may be the reason for the insignificant difference in severe CAC score (Agatston score > 400) between the two groups. Another interesting finding is that there is a significant correlation between HDL and CAT when female patients are analyzed independently. In this way, previous hypothesis that there was no significant difference between both groups regarding HDL level was overturned by us. HDL is known to exert an anti-atherosclerotic effect by interacting with macrophages and other inflammatory immune cells [27]. As other studies have shown that CAT is inversely associated with atherosclerosis [8]. Perhaps a high level of HDL may be a potential protection mechanism.

### Limitations

This study is a single-center cross-sectional study, which limits the comparability of our findings to the general population. In addition, there is a lack of classification in CAT severity, which is uniformly defined as at least three consecutive curvatures greater than 45 degrees in a major epicardial coronary artery. Moreover, there may be some differences between the two methods of coronary angiography and CTA in the assessment of coronary artery stenosis.

## Conclusions

Tortuous arteries are common in humans. Although slight tortuosity is asymptomatic, severe tortuosity may cause an ischemic attack in remote organs. Our results show that tortuous arteries are related to aging, hypertension, BMI, and gender. However, little is known about the mechanism of its formation and development. In the future, more research is needed to explore the mechanism and provide new technologies to prevent and treat vascular curvature.

## Supplementary Information

**Additional file 1.** Univariable analysis of variables associated with CAT.

## Data Availability

All data are available without restriction. Researchers can obtain data by contacting the corresponding author.

## Abbreviations

CAT: Coronary artery tortuosity
CAC: Coronary artery calcification
OR: Odds ratio
95%CI: Confidence interval
BMI: Body mass index
CTA: Computed tomography angiography
AO: Aortic sinus
AAO: Ascending aorta
LA: Left atrial anteroposterior diameter
LVEDd or RVEDd: Left or right ventricular end-diastolic inner diameter
LVPW: Left ventricular posterior wall thickness
IVS: Interventricular septal thickness
LVEF: Left ventricular ejection fraction
TG: Triglycerides
HDL: High density lipoprotein
ApoA1: Apolipoprotein A1
ApoB: Apolipoprotein B
LPa: Lipoprotein a
