# Supplementary material for: Correlation analysis of coronary artery tortuosity and calcification score
Source: BMC Surg. 2022 Feb 23;22:66. doi: 10.1186/s12893-022-01470-w (PMC8867736; doi:10.1186/s12893-022-01470-w)
Supplement: Supplementary file 1 — Additional file 1. Univariable analysis of variables associated with CAT. [file 12893_2022_1470_MOESM1_ESM.docx]

Supplementary Table 1. Univariable analysis of which factors are related to CAT

|  | Univariable analysis | |
| --- | --- | --- |
| Variable | Odds ratio (95% CI) | *p*-value |
| Gender |  |  |
| Male(n) | Ref |  |
| Female (n) | 1.92 (1.52-2.42) | <0.001 |
| Age (years) | 1.03 (1.02-1.04) | <0.001 |
| BMI (kg/m^2^) |  |  |
| 18.5-23.9 | Ref |  |
| 24-27.9 | 0.71 (0.55-0.92) | 0.009 |
| ＞28 | 0.41 (0.29-0.59) | <0.001 |
| Myocardial bridge | 1.20 (0.95-1.51) | 0.122 |
| Diameter stenosis |  |  |
| No stenosis | Ref |  |
| Slight stenosis | 1.15 (0.82-1.61) | 0.434 |
| Mild stenosis | 1.22 (0.89-1.68) | 0.218 |
| Moderate stenosis | 1.47 (1.04-2.09) | 0.029 |
| Severe stenosis | 0.96 (0.63-1.45) | 0.837 |
| Agatston score |  |  |
| 0 | Ref |  |
| 1-100 | 1.07 (0.81-1.42) | 0.633 |
| 101-400 | 1.49 (1.05-2.10) | 0.025 |
| ＞400 | 1.29 (0.89-1.86) | 0.17 |
| Smoking | 0.81 (0.58-1.12) | 0.203 |
| Hypertension | 1.34 (1.06-1.70) | 0.015 |
| Diabetes | 0.91 (0.68-1.21) | 0.517 |
| Biochemical indexes |  |  |
| Uric Acid (umol/l) | 0.99 (0.97-1.00) | 0.019 |
| TG (mmol/l) | 0.90 (0.81-1.00) | 0.049 |
| CHOL (mmol/l) | 1.04(0.93-1.16) | 0.475 |
| HDL (mmol/l) | 2.02 (1.38-2.96) | <0.001 |
| LDL (mmol/l) | 0.95 (0.82-1.10) | 0.496 |
| ApoA1(g/l) | 1.13 (0.73-1.76) | 0.582 |
| ApoB (g/l) | 0.89 (0.54-1.45) | 0.633 |
| LPa (mg/l) | 1.00 (1.00-1.00) | 0.067 |

Supplementary Table 2. Univariable analysis of variables associated with CAT in different gender

|  | Univariable analysis (female) | | Univariable analysis (male) | |
| --- | --- | --- | --- | --- |
| Variable | Odds ratio (95% CI) | *p*-value | Odds ratio (95% CI) | *p*-value |
| Age (years) | 1.03 (1.01-1.04) | <0.001 | 1.02 (1.00-1.03) | 0.002 |
| BMI (kg/m2) |  |  |  |  |
| 18.5-23.9 | Ref |  | Ref |  |
| 24-27.9 | 0.75 (0.53-1.07) | 0.111 | 0.77 (0.52-1.13) | 0.175 |
| ＞28 | 0.49 (0.31-0.81) | 0.005 | 3.62 (0.21-0.64) | <0.001 |
| Myocardial bridge | 1.17(0.85-1.62) | 0.329 | 1.27(0.90-1.79) | 0.172 |
| Diameter stenosis |  |  |  |  |
| No stenosis | Ref |  | Ref |  |
| Slight stenosis | 0.72(0.38-1.36) | 0.311 | 1.61(0.98-2.64) | 0.060 |
| Mild stenosis | 0.71(0.34-1.47) | 0.357 | 1.67(1.02-2.74) | 0.041 |
| Moderate stenosis | 0.74(0.37-1.48) | 0.394 | 1.51(0.90-2.52) | 0.116 |
| Severe stenosis | 1.39(0.66-2.94) | 0.383 | 1.01(0.56-1.82) | 0.937 |
| Agatston score |  |  |  |  |
| 0 | Ref |  | Ref |  |
| 1-100 | 1.49 (1.01-2.18) | 0.042 | 0.79 (0.51-1.21) | 0.278 |
| 101-400 | 2.36 (1.36-4.07) | 0.002 | 1.26 (0.78-2.03) | 0.347 |
| ＞400 | 1.82 (1.07-3.11) | 0.028 | 1.07 (0.64-1.81) | 0.789 |
| Smoking | 1.37(0.34-5.5) | 0.658 | 1.16(0.79-1.69) | 0.443 |
| Hypertension | 1.68 (1.21-2.33) | 0.002 | 1.08 (0.76-1.54) | 0.660 |
| Diabetes | 1.04(0.69-1.58) | 0.846 | 0.90(0.60-1.35) | 0.620 |
| Biochemical indexes |  |  |  |  |
| Uric Acid (umol/l) | 1.00 (0.99-1.00) | 0.452 | 1.00 (0.99-1.00) | 0.104 |
| TG (mmol/l) | 0.89 (0.77-1.06) | 0.191 | 0.93 (0.81-1.07) | 0.323 |
| CHOL (mmol/l) | 1.00(0.88-1.19) | 0.799 | 0.98(0.83-1.15) | 0.800 |
| HDL (mmol/l) | 1.88 (1.12-3.18) | 0.018 | 1.38 (0.76-2.50) | 0.290 |
| LDL (mmol/l) | 0.93(0.75-1.16) | 0.542 | 0.93(0.75-1.15) | 0.479 |
| ApoA1(g/l) | 0.83(0.46-1.52) | 0.546 | 1.06(0.53-2.10) | 0.874 |
| ApoB (g/l) | 1.3(0.66-2.60) | 0.437 | 0.58(0.28-1.20) | 0.141 |
| LPa (mg/l) | 1.00(1.00-1.00) | 0.097 | 1.00(1.00-1.00) | 0.432 |
